# The Bacteria‐Fungi‐Phage Interplay in Periodontitis and Peri‐Implantitis

**DOI:** 10.1155/ijod/7239600

**Published:** 2026-08-02

**Authors:** Zhaoxin Ji, Lingying Kang, Sirui Liu, Qingsong Jiang, Wei Wei

**Affiliations:** ^1^ Beijing Stomatological Hospital, Capital Medical University, Beijing, China, ccmu.edu.cn

**Keywords:** bacteria–fungi interactions, dysbiosis, oral microbiome, oral mycobiome, peri-implantitis, periodontitis

## Abstract

**Objective:**

This review aims to summarize current evidence on the interactions among bacteria, fungi, and bacteriophages in periodontitis and peri‐implantitis, and to discuss their ecological significance, pathogenic mechanisms, and potential clinical implications.

**Subjects and Methods:**

This review synthesizes current insights into the roles of the oral microbiome in these diseases, with a focus on the critical interplay between bacteria, fungi, and bacteriophages.

**Results:**

Our analysis demonstrates that disease progression is marked by a shift toward polymicrobial synergy. Keystone pathogens and opportunistic fungi engage in intricate interactions within biofilms, including physical coadhesion and metabolic cross‐feeding, which enhance microbial resilience and virulence. Bacteriophages, acting as natural modulators of bacterial populations, emerge as a promising therapeutic approach to disrupt these pathogenic communities. This bacteria‐fungi‐phage consortium synergistically modulates host immune responses, fostering chronic inflammation and tissue destruction.

**Conclusion:**

An integrated, multikingdom perspective on the oral ecosystem is critical for clinical advancement. Future strategies should prioritize personalized interventions that combine multiomics biomarker analysis with targeted therapies to effectively disrupt polymicrobial biofilms, restore homeostasis, and overcome antimicrobial resistance.

## 1. Introduction

As the most prevalent chronic inflammatory diseases in the oral cavity, periodontitis and peri‐implantitis are the primary drivers of natural tooth and implant loss. Periodontitis is a plaque biofilm‐induced chronic inflammatory disease that affects the tooth‐supporting tissues, including the gingiva, periodontal ligament, cementum, and alveolar bone. It is characterized by gingival inflammation, periodontal pocket formation, clinical attachment loss, and progressive alveolar bone resorption, ultimately leading to tooth mobility and tooth loss if left untreated [1]. The impact of these conditions is substantial and far‐reaching, creating a health burden and a cascade of economic consequences that present a pressing global challenge. Findings from the Global Burden of Disease Study point to periodontitis as a major global health concern, afflicting close to 10% of humanity and ranking it as the sixth most common disease worldwide [2]. Peri‐implantitis is a plaque‐associated pathological condition occurring in tissues around dental implants, characterized by inflammation of the peri‐implant mucosa and progressive loss of supporting bone [3]. It affects 20%–45% of implant recipients and remains a leading cause of implant failure [4]. Despite extensive research and established treatments, both diseases remain major causes of oral rehabilitation failure, highlighting the need for a deeper understanding of their microbial underpinnings.

Through the lens of a bacterium‐centric paradigm, research has consistently pointed to a critical consortium of pathogens—notably *Porphyromonas gingivalis*, *Tannerella forsythia*, and *Treponema denticola*—as key drivers of disease etiology [5]. While this paradigm has profoundly shaped our understanding of bacterial dysbiosis and host‐microbe dynamics, it is now confronted with significant limitations. The bacterial model alone cannot fully explain the marked heterogeneity in disease progression nor why certain individuals exhibit tissue breakdown despite similar bacterial profiles. Moreover, therapies exclusively targeting bacteria often yield inconsistent clinical outcomes, suggesting that crucial ecological factors have been overlooked.

Recent advances in microbial ecology have fundamentally redefined our perspective: the focus has moved beyond individual pathogens to a polymicrobial synergy and dysbiosis model. This new paradigm posits that disease emerges from complex cooperative interactions across diverse microbial kingdoms. Within this context, the oral mycobiome has emerged as a key yet underappreciated player. *Candida albicans* (*C. albicans*), though a commensal organism, forms intimate partnerships with bacteria in mixed‐species biofilms, where it enhances bacterial adhesion, provides anaerobic niches, and amplifies inflammation [6–8]. Such cross‐kingdom cooperation not only strengthens biofilm resilience but also modulates host immunity, accelerating tissue destruction and contributing to treatment resistance. Adding another layer of complexity, bacteriophages represent a third major biological force shaping the oral ecosystem [9]. Far from being passive residents, phages actively modulate bacterial population dynamics, horizontal gene transfer, and virulence factor expression [10]. This dual role makes them a compelling target: one that promises to yield profound mechanistic insights and enable precise clinical interventions in the management of these complex diseases.

Despite these advances, a significant gap remains between cutting‐edge microbiome discoveries and their integration into a coherent clinical framework for managing oral inflammatory diseases. This review critically evaluates current evidence on the interplay among bacteria, fungi, and bacteriophages in the oral microbiome associated with periodontitis and peri‐implantitis.

## 2. The Oral Bacteriome in Periodontitis and Peri‐Implantitis

### 2.1. The Healthy Oral Bacteriome

The oral microbiome comprises bacteria, fungi, viruses, and archaea. Among these microbial communities, the bacteriome constitutes the dominant component and plays a central role in maintaining oral ecological homeostasis [11]. The dominant phyla in a healthy oral microbiome are *Firmicutes*, *Bacteroidetes*, *Actinobacteria*, and *Proteobacteria*, with *Streptococcus*, *Veillonella*, *Prevotella*, and *Actinomyces* being the most abundant genera [12, 13]. The healthy microbiome maintains a state of homeostasis, where beneficial microorganisms inhibit the growth of harmful pathogens through mechanisms such as competitive exclusion and antimicrobial peptide secretion [14].

### 2.2. The Bacteriome in Periodontitis

Dysbiosis, the microbial imbalance that occurs in periodontal disease, is characterized by an increase in the abundance of pathogenic bacteria, which disrupt the balance of the microbial community. In periodontitis, the microbial composition shifts towards a dominance of red‐complex bacteria, including *Porphyromonas gingivalis*, *Tannerella forsythia*, and *Treponema denticola*, which are considered the keystone pathogens driving disease progression [15]. Recent studies utilizing 16S rRNA gene sequencing have provided insights into the complex microbial shifts that occur during periodontitis. 16S rRNA sequencing studies have shown that periodontitis and peri‐implantitis are associated with an altered abundance of anaerobic Gram‐negative taxa, including *Porphyromonas*, *Tannerella*, and *Treponema*, accompanied by reduced microbial diversity and disruption of ecological balance [16]. Similar dysbiotic shifts have been reported around diseased implants, although the relative abundance of specific taxa may differ from that observed in periodontitis [17]. Inflammatory cytokines such as tumor necrosis factor‐alpha (TNF‐α), interleukin‐1 beta (IL‐1β), and interleukin‐6 (IL‐6) are upregulated in response to the increased abundance of pathogenic bacteria, exacerbating tissue destruction and periodontal pocket formation [18]. Integrating state‐of‐the‐art high‐throughput microbial sequencing techniques with function‐based analytical methods enables a more comprehensive and high‐resolution perspective for microbiome reconstruction in periodontitis [19].

### 2.3. The Bacteriome in Peri‐Implantitis

Peri‐implantitis, a condition characterized by inflammation around dental implants, shares many similarities with periodontitis in terms of microbial composition. However, the bacteriome in peri‐implantitis is often more diverse and complex due to the distinct surface characteristics of dental implants and the unique microenvironment they create. Studies have shown that the microbiome around implants is characterized by a higher prevalence of anaerobic and Gram‐negative bacteria, including *P. gingivalis*, *Fusobacterium nucleatum*, and *Prevotella intermedia*, which are also associated with periodontitis. Furthermore, the immune response associated with peri‐implantitis was found to exhibit a skewed pattern, with a dominant presence of Th2 and Th17 cell subsets [20]. 16S rRNA sequencing and metagenomic analyses of peri‐implant biofilms have revealed that pathogenicity islands and virulence genes from these microbiomes are enriched in peri‐implantitis samples [21], leading to greater difficulty in achieving effective treatment and implant survival. 16S rRNA sequencing studies have shown that peri‐implantitis is associated with a more diverse and heterogeneous microbial community than periodontitis [17]. Besides classical periodontal pathogens, peri‐implant lesions frequently harbor opportunistic species such as *Staphylococcus*, *Pseudomonas*, and *Enterococcus*. Moreover, several studies have reported increased fungal colonization and a greater prevalence of nonoral microorganisms, suggesting that peri‐implantitis may represent a distinct ecological niche with unique microbial interactions [22].

## 3. The Oral Mycobiome in Periodontitis and Peri‐Implantitis

### 3.1. The Healthy Oral Mycobiome

The oral mycobiome represents the fungal component of the oral microbiome and constitutes a minor but indispensable part of the oral microbial ecosystem [23]. In healthy individuals, *Candida* species are predominant, followed by *Cladosporium*, *Aureobasidium*, *Aspergillus*, *Fusarium*, and *Cryptococcus*. Ghannoum et al. [24] first established the concept of an oral “mycobiome” by internal transcribed spacer (ITS)‐based sequencing, demonstrating that fungi from both the *Ascomycota* and *Basidiomycota* phyla coexist with commensal bacteria to maintain homeostasis. Subsequent studies confirmed that in healthy individuals, fungal diversity remains relatively stable across sex and age, with *Candida*, *Malassezia*, and *Saccharomyces* being the dominant genera [25].

### 3.2. The Mycobiome in Periodontitis

Periodontitis is characterized by a dysbiotic shift in both bacterial and fungal communities. Although substantial interindividual variability exists, studies have reported shifts in the composition of the oral mycobiome in periodontitis, indicating that fungal communities may contribute to disease‐associated microbial dysbiosis. Among the fungal taxa detected in periodontal sites, *Candida* species are frequently identified and have attracted increasing attention because of their potential role in oral biofilm development and host–microbe interactions [7, 26]. In vitro and clinical data suggest that *C. albicans* forms synergistic biofilms with *Porphyromonas gingivalis*, enhancing bacterial adhesion and inflammation [23]. The hyphal form of *C. albicans* demonstrates higher invasive capability and elicits stronger epithelial immune responses, contributing to tissue destruction and chronicity [27].

### 3.3. The Mycobiome in Peri‐Implantitis

Fungal colonization is more prevalent in peri‐implantitis compared with healthy implants. A systematic review found *C. albicans*, *C. parapsilosis*, and *C. tropicalis* to be the most common species associated with peri‐implant infections [28]. Quantitative polymerase chain reaction (qPCR) analyses have revealed higher fungal loads in peri‐implant sulcus fluid, inside implant‐abutment connections, and in adjacent gingival sites [29]. The presence of *Candida* biofilms on implant surfaces may facilitate bacterial adhesion and contribute to a proinflammatory microenvironment, potentially aggravating peri‐implant tissue destruction. These findings suggest that fungal colonization may contribute to biofilm maturation, microbial persistence, and inflammatory tissue destruction around the implants.

Overall, the eukaryotic nature of fungi fundamentally distinguishes them from prokaryotic bacteria, endowing them with an enhanced capacity to activate the host immune system. Moreover, their ability to undergo morphological transitions into invasive hyphae allows fungi to penetrate host tissues, further contributing to their pathogenic potential [30].

## 4. The Bacteriophages in Periodontitis and Peri‐Implantitis

Bacteriophages (phages) are viruses that specifically target and infect bacteria, playing a crucial role in regulating bacterial populations within the oral microbiome. Their capacity to disrupt biofilms is a key therapeutic attribute, enhancing effectiveness against diverse oral pathogens in periodontitis, peri‐implantitis, and apical periodontitis [31]. In healthy individuals, phages help maintain microbial balance by controlling bacteria implicated in periodontal diseases [32].

**Figure 1:**
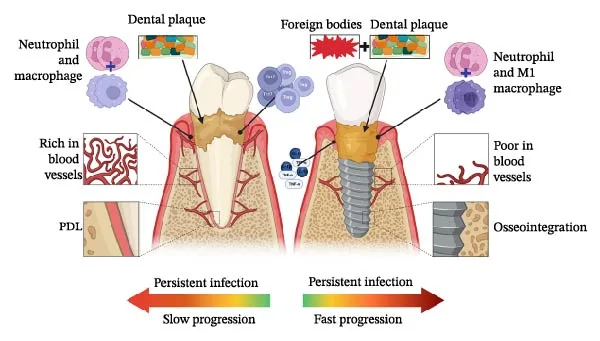
Comparative ecological landscape of periodontitis and peri‐implantitis. This figure compares the anatomical and immune differences between periodontitis (left) and peri‐implantitis (right). Natural teeth are characterized by the presence of a periodontal ligament, rich vascularization, and perpendicular collagen fiber insertion, whereas implants lack a periodontal ligament and exhibit reduced vascular supply and parallel collagen fiber orientation. These anatomical differences influence microbial colonization, host immune responses, and biofilm development.

Importantly, although periodontitis and peri‐implantitis share overlapping microbial dysbiosis profiles, phage behavior and therapeutic performance may differ between these two disease environments due to ecological and structural conditions [33] (Figure 1). In periodontitis, phages act primarily within periodontal pockets that are vascularized and fluid‐dynamic, where the presence of periodontal ligament‐associated vascular networks facilitates immune cell trafficking and interstitial fluid exchange, thereby supporting diffusion‐based microbial targeting [34]. In contrast, peri‐implantitis develops on implant‐associated biofilms formed on titanium or zirconia surfaces, where the absence of the periodontal ligament, reduced vascular supply, and distinct collagen fiber orientation create a structurally and immunologically different microenvironment [35, 36]. These differences suggest that peri‐implant environments may impose additional biophysical constraints on phage–biofilm interactions compared with natural periodontal tissues, particularly in terms of microbial adhesion strength and biofilm structural stability, which are known to influence phage diffusion and biofilm penetration efficiency in established phage–biofilm models [37].

The potential of phage therapy has been highlighted by recent in vitro studies, making it a promising approach for treating periodontitis, particularly cases involving antimicrobial resistance. However, confirming these results requires further studies in more complex environments, such as multispecies biofilms and clinical applications [38].

Bacteriophages not only interact with bacterial populations but may also indirectly affect fungal dynamics in the oral microbiome. Recent studies suggest that phages might be engineered to display fungal antigens on their surfaces, triggering immune responses such as activating Th1 and Th17 pathways or producing neutralizing antibodies. Additionally, combining phages with photodynamic inactivation or antimicrobials could enhance treatment efficacy. Experimental studies suggest that phage‐based platforms may exert indirect or engineered antifungal effects [39].

Promising results have also been observed in using phage therapy as an adjunct treatment for periprosthetic joint infections caused by *Staphylococcus aureus*, especially when conventional antibiotics are challenged by resistance [40]. Moreover, bacteriophages and their derived enzymes can effectively target bacterial biofilms in infections like those in the periodontium and around implants. Especially when combined with antibiotics, they show promise in improving biofilm removal in various models [41, 42]. Based on the current research progress, phage therapy demonstrates considerable potential for clinical translation in the prevention and treatment of periodontal and peri‐implant diseases.

## 5. Interactions Among Microbial Communities and With the Host in Oral Dysbiosis

The nature of microbial interactions is strongly influenced by the ecological environment in which biofilms develop. Compared with periodontal tissues, peri‐implant sites provide distinct ecological conditions, including differences in surface characteristics, nutrient availability, and host–microbe interfaces. These factors may contribute to the formation of more complex dysbiotic communities and help explain the often more rapid and aggressive progression of peri‐implantitis. Understanding these ecological influences is therefore essential for interpreting bacteria–fungi–phage interactions in oral inflammatory diseases. Bacteria and fungi coexist within the oral cavity, engaging in a complex network of mutualistic and competitive interactions that shape dysbiotic microbial communities. These cross‐kingdom relationships influence not only microbial survival and virulence but also the host immune responses that determine disease progression in periodontitis and peri‐implantitis.

### 5.1. Physical Interactions

Direct physical contact facilitates bacterial adhesion to fungal surfaces, enabling structural cooperation in mixed biofilms [43]. *Porphyromonas gingivalis* has been shown to adhere to *C. albicans* hyphae through protein–protein interactions mediated by *InlJ* and *Als3*, establishing a stable “transportation network” that promotes biofilm maturation and microbial persistence under stress [44]. Such coadhesion increases bacterial survival in aerobic conditions and enhances biofilm resilience against mechanical disruption and antimicrobial agents [45].

### 5.2. Metabolic Interactions

Metabolic crosstalk is a cornerstone of bacteria–fungi synergy; the metabolic processes of bacteria can alter their microenvironment and continuously impact the survival and growth of neighboring species [46]. Bacterial metabolites such as lactate can serve as carbon sources for *C. albicans*, while fungal oxygen consumption generates microanaerobic niches that favor the growth of strict anaerobes like *P. gingivalis*—a phenomenon described as cross‐kingdom feeding [47]. Recent multiomics investigations have further elucidated that in *Streptococcus mutans–C. albicans* mixed‐species biofilms, the fungal partner undergoes extensive metabolic reprogramming facilitated by *S. mutans*‐derived glucosyltransferase B [48]. Moreover, heme competition within the mixed biofilm environment has been found to increase the pathogenic potential of *P. gingivalis*, leading to enhanced virulence and tissue destruction [49].

### 5.3. Quorum Sensing and Molecular Communication

Interkingdom communication via quorum‐sensing (QS) molecules represents another key mechanism of coordination. Fungal QS molecules such as farnesol and tyrosol can modulate bacterial biofilm formation and gene expression, while bacterial autoinducers may influence fungal morphogenesis [50]. Although extensively studied in bacterial communities, the full spectrum of QS‐like signaling between bacteria and fungi in the oral cavity remains underexplored, representing an emerging research frontier [51].

### 5.4. Synergistic Virulence and Host Immune Modulation

The coexistence of bacteria and fungi often amplifies pathogenicity through synergistic immune evasion strategies. Clinically, coinfection with these organisms correlates with active periodontitis and enhanced inflammatory tissue destruction [52]. The complex interactions between bacteria and fungi can mask bacterial antigens from host recognition, triggering both innate and adaptive immune responses that shape the progression of diseases such as periodontitis and peri‐implantitis [53]. Epithelial cells act as the first line of defense, expressing pattern recognition receptors (PRRs) such as Toll‐like receptor 2 (TLR2) and Toll‐like receptor 4 (TLR4), which recognize microbial components such as lipopolysaccharides (LPS) and fungal cell wall components like β‐glucans. Activation of these receptors leads to the initiation of proinflammatory signaling cascades, including the nuclear factor kappa B (NF‐κB) pathway [54]. In the presence of mixed bacterial and fungal biofilms, neutrophil function can be impaired, and the formation of neutrophil extracellular traps (NETs) may be enhanced within biofilms, promoting tissue damage and increasing inflammation [55]. In oral dysbiosis, macrophages are often skewed toward the M2 phenotype in mixed‐species biofilms, leading to chronic inflammation [56]. Dysregulation of the balance between these pro‐ and anti‐inflammatory cytokines can also lead to either chronic inflammation or tissue damage in periodontal and peri‐implant tissues. Thus, the host immune system perceives the bacterial–fungal consortium not as separate pathogens but as a composite, partially camouflaged entity capable of chronic immune modulation.

**Figure 2:**
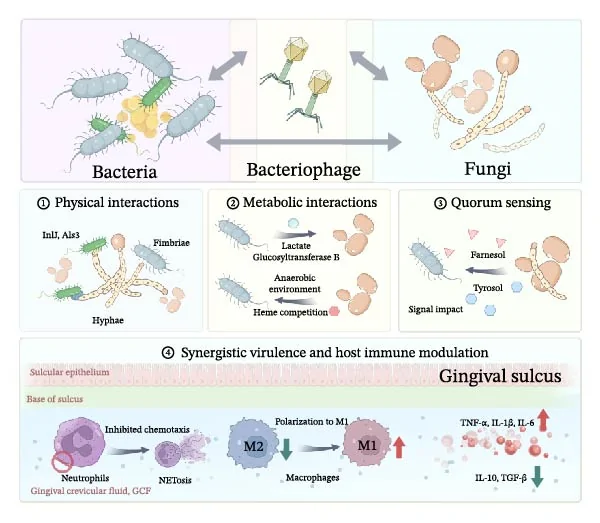
Schematic representation of the bacteria–fungi–host immune interaction network in oral dysbiosis. This schematic illustrates the interactions between bacteria, fungi, and the host immune system in oral dysbiosis. Bacteria and fungi interact through physical adhesion, metabolic exchange, and quorum sensing. The host immune response involves neutrophil suppression, macrophage polarization, and changes in the cytokine network, promoting inflammation and altering immune balance.

Overall, the bacteria–fungi–host triad forms a dynamic and reciprocal system (Figure 2). Physical and metabolic cooperation within the biofilm creates a protective niche, while signaling crosstalk and immune evasion mechanisms sustain chronic inflammation. Deciphering these complex interkingdom interactions will be crucial for understanding oral dysbiosis and designing targeted microbiome‐based therapeutic strategies.

## 6. Clinical Implications

Current diagnostic approaches that rely on detecting a limited set of bacterial species fall short of capturing the full complexity of oral diseases. Although bacterial biomarkers derived from methods such as 16S rRNA sequencing offer valuable insights into microbial dysbiosis, they often overlook the role of fungi in disease development. Fungal infections are known to exacerbate biofilm‐related diseases yet remain frequently undetected in routine diagnostics. Advances in sequencing technology, such as ITS‐based methods, now enable the integration of fungal biomarkers into diagnostic workflows. Combining bacterial and fungal biomarkers has shown clinical value: robust machine learning models demonstrate improved prediction of coinfections [57]. For high‐risk individuals with periodontitis or peri‐implantitis, integrating both bacterial and fungal microbiome data could enhance diagnostic accuracy and support earlier, more targeted treatments.

Another growing concern is the overuse of antibiotics, which disrupts microbial balance and encourages the proliferation of opportunistic fungi like *Candida*. This highlights the need for more balanced treatment approaches that incorporate both antibacterial and antifungal agents. In peri‐implantitis, combining mechanical debridement with localized antifungal agents has proven effective in disrupting polymicrobial biofilms and controlling inflammation. Such integrated approaches not only target bacteria but also curb fungal overgrowth, helping restore ecological equilibrium. Clinically, this may involve supplementing traditional antibiotic regimens with short‐term antifungal courses to limit fungal recurrence, such as fluconazole or nystatin mouthwash. However, challenges persist in establishing clear pathogenic thresholds and differentiating colonization from active infection, underscoring the need for multidisciplinary validation [58].

Beyond conventional antimicrobial approaches, microbiome‐based therapies are emerging as a promising strategy for the management of periodontitis and peri‐implantitis. Unlike traditional treatments that primarily aim to eliminate pathogenic microorganisms, these approaches seek to restore microbial homeostasis and rebalance dysbiotic communities. Recent studies have highlighted several microbiome‐based therapeutic modalities, including probiotics, postbiotics, predatory bacteria, and bacteriophages, as well as microbiota transplantation. These strategies may modulate microbial composition, suppress pathogenic taxa, enhance beneficial microbial functions, and promote host immune regulation. Given the growing recognition that periodontitis and peri‐implantitis are driven by polymicrobial dysbiosis rather than individual pathogens, microbiome‐based interventions may provide a more ecological and sustainable therapeutic approach. Nevertheless, most microbiome‐based therapies remain at the preclinical or early translational stage, and further studies are required to establish their long‐term efficacy, safety, and clinical applicability [59].

## 7. Future Directions

The progression of periodontal disease and the imbalance of peri‐implant tissue homeostasis are closely tied to abnormalities in the oral microbial community [60, 61]. Traditional microbial studies, primarily based on 16S rRNA and ITS sequencing, have provided insights into the presence of pathogenic bacteria and fungi but fail to explain the functional roles of these microbial communities [62, 63]. The integration of multiomics technologies has enabled the shift from identifying “who is there” to understanding “what they are doing.” Transcriptomics reveals gene expression in microbial communities, linking genetic potential to functional output, while proteomics provides insights into microbial proteins and host–microbe interactions. Metabolomics connects microbial metabolic activities to host health by analyzing metabolites like short‐chain fatty acids and LPS [64]. These technologies have provided new understanding of periodontal and peri‐implant diseases, including the role of gene‐metabolite‐pathway regulation in periodontitis [65] and implant material interactions in peri‐implantitis [66].

**Figure 3:**
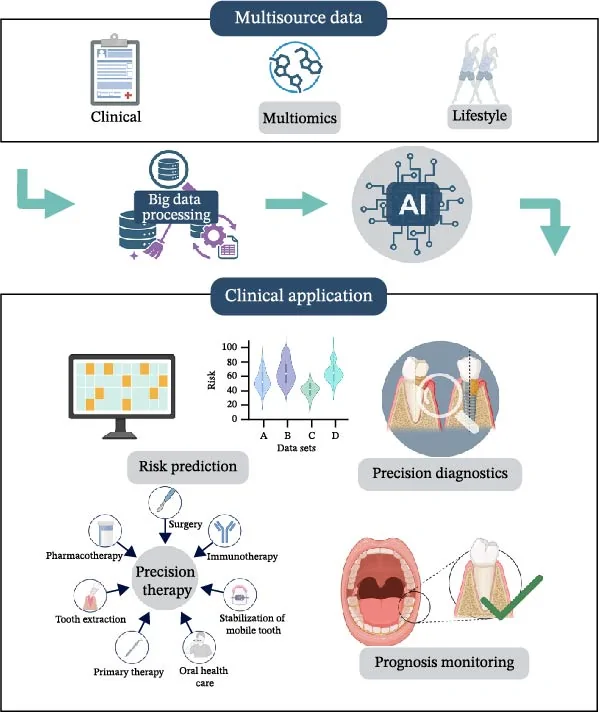
AI and multiomics integration for studying bacteria–fungi–phage interactions. AI and big data enable the integration of multiomics, clinical, and lifestyle datasets to uncover complex bacteria–fungi–host interactions in periodontitis and peri‐implantitis. These approaches facilitate risk prediction, precision diagnostics, and personalized therapeutic strategies.

Advances in multiomics technologies are generating increasingly complex datasets that capture the composition and interactions of bacterial, fungal, and phage communities within the oral microbiome. In this context, artificial intelligence (AI) and machine learning approaches are emerging as powerful tools for integrating and interpreting these high‐dimensional datasets (Figure 3). Recent studies have demonstrated the utility of AI‐driven models in microbiome‐based disease prediction, biological aging assessment, and identification of antibiotic resistance genes [67]. More importantly, AI‐based analytical frameworks can uncover microbial interaction networks and identify key taxa associated with dysbiosis, thereby providing new insights into the ecological mechanisms underlying periodontitis and peri‐implantitis. Combined with emerging technologies such as hyperspectral imaging and multiomics profiling, AI may facilitate the development of precision diagnostics and personalized therapeutic strategies based on individual microbial signatures [68, 69]. As our understanding of bacteria–fungi–phage interactions continues to expand, AI‐assisted analyses are expected to play an increasingly important role in translating complex microbiome data into clinically actionable knowledge.

## 8. Conclusion

The interplay among bacterial, fungal, and phage communities within the oral microbiome critically influences the progression of periodontitis and peri‐implantitis. Both bacterial and fungal communities contribute synergistically to the dysbiosis seen in these conditions, intensifying inflammatory responses and accelerating tissue damage. Although conventional therapies predominantly target bacteria, growing evidence supports a more integrated approach that addresses both microbial kingdoms in the diagnosis and treatment. Combining bacteriophage therapy with standard interventions presents a promising avenue for disrupting complex biofilms and modulating inflammation. Future studies should validate these strategies within multispecies biofilm environments and through well‐designed clinical trials. Ultimately, high‐throughput sequencing and microbiome‐guided frameworks are poised to transform the management of periodontal and peri‐implant diseases, enabling more precise and individualized therapeutic solutions. Further elucidation of bacteria–fungi–phage interactions through multiomics and AI‐assisted analyses may facilitate the development of next‐generation microbiome‐targeted therapies for periodontal and peri‐implant diseases.

## Author Contributions

Wei Wei and Qingsong Jiang conceived the study. Zhaoxin Ji wrote the first draft with assistance from Wei Wei and Qingsong Jiang. Sirui Liu helped draw Figure 1. Lingying Kang helped draw Figures 2 and 3.

## Funding

This work was supported by the National Natural Science Foundation of China (Grant 82501147), the Beijing Stomatological Hospital, Capital Medical University Young Scientist Program (Grant YSP202314), the Capital Health Development Scientific Research Special Project (Grant 2022‐1‐2141), and the Beijing Municipal Hospital Management Center “Yangfan” Initiative (Grant ZLRK202530).

## Disclosure

All authors have critically reviewed and approved the final manuscript.

## Consent

The authors have nothing to report.

## Conflicts of Interest

The authors declare no conflicts of interest.

## Data Availability

Data sharing is not applicable to this article as no datasets were generated or analyzed during the current study.
